# The updated South African National Guideline for the Prevention of Mother to Child Transmission of Communicable Infections (2019)

**DOI:** 10.4102/sajhivmed.v21i1.1079

**Published:** 2020-07-08

**Authors:** Jeannette Wessels, Gayle Sherman, Lesley Bamford, Manala Makua, Mathilda Ntloana, James Nuttall, Yogan Pillay, Ameena Goga, Ute Feucht

**Affiliations:** 1 Research Centre for Maternal, Fetal, Newborn and Child Health Care Strategies, University of Pretoria, Pretoria, South Africa; 2 Department of Paediatrics & Child Health, Faculty of Health Sciences, University of the Witwatersrand, Johannesburg, South Africa; 3 Centre for HIV & STI, National Institute for Communicable Diseases, Division of the National Health Laboratory Services, Johannesburg, South Africa; 4 Child, Youth, and School Health Chief Directorate, National Department of Health, Pretoria, South Africa; 5 School of Health Systems and Public Health, University of Pretoria, Pretoria, South Africa; 6 Communicable and Non-Communicable Diseases Branch, National Department of Health, Pretoria; 7 Department of Paediatrics and Child Health, Red Cross War Memorial Children’s Hospital, University of Cape Town, Cape Town, South Africa; 8 Health Systems Research Unit, South African Research Council, Cape Town, South Africa; 9 Department of Paediatrics and Child Health, University of Pretoria, Pretoria, South Africa; 10 HIV Prevention Research Unit, South African Medical Research Council, Cape Town, South Africa; 11 Tshwane District Health Services, Gauteng Department of Health, Tshwane, South Africa; 12 Maternal and Infant Health Care Strategies Research Unit, South African Medical Research Council, Pretoria, South Africa

## Introduction

South Africa has made great strides in reducing the vertical transmission of human immunodeficiency virus (HIV) in the first two months of life from 23% (2003) to 0.7% (2019), despite a persistently high antenatal HIV prevalence of around 30%. Improving access to antiretroviral therapy during antenatal care has significantly contributed to this success, but has led to an increase in the relative proportion of vertical transmissions due to breastfeeding in the first six months post-delivery. Yet, due to the short- and long-term benefits of breastfeeding and risks associated with not breastfeeding, mothers need to be supported to breastfeed their infants for the longest duration possible, while maintaining virological suppression to reduce the vertical transmission risk.

The new South African National Guideline for the Prevention of Mother to Child Transmission of Communicable Infections (2019) outlines three major strategies for programme improvement. These are 1) prevention of primary HIV infection and unintended pregnancies in women of childbearing potential, 2) improvement of maternal viral suppression rates at delivery and in the post-delivery period through potent, well-tolerated antiretroviral regimens, strategic use of maternal viral load monitoring, linking of mothers to post-delivery HIV care and integration of mother-infant health care, and 3) provision of enhanced prophylaxis to infants of mothers with elevated HIV viral loads in the breastfeeding period, while every effort is made to regain maternal viral suppression.

Rigorous implementation of this guideline can potentially move South Africa closer to the goal of eliminating mother-to-child transmission and making an HIV-free generation a reality.

South Africa’s (SA) programme of prevention of mother-to-child transmission (PMTCT) of HIV has achieved remarkable successes in recent years in ensuring good outcomes for pregnant women living with HIV and reducing the risk of vertical HIV transmission to their children.^1^ The most critical intervention to prevent vertical transmission is the maintenance of the undetectable maternal HIV viral load (VL) levels through effective antiretroviral therapy (ART).^2^ This has necessitated a considerable scale-up of HIV testing services (HTS) within antenatal care to identify women living with HIV (WLWH). The PMTCT ‘Option B Plus’, namely the provision of lifelong ART irrespective of CD4 count or clinical disease severity, was implemented in South Africa in January 2015, and significantly improved access to ART for pregnant women in the public sector.^3,4^ As a result, more than 95% of women with unknown HIV status are currently tested for HIV during antenatal care, and more than 90% of WLWH are on ART.^5^ Vertical transmission rates within the first two months of life dropped dramatically from 23% in 2003^6^ to 0.7% in 2019.^7^

Whilst the achievements in reducing HIV transmission at birth are noteworthy, the Global Plan target^1^ of *elimination* of vertical transmission will remain elusive due to SA’s high HIV prevalence rates. This brings into stark focus the need for primary prevention of HIV in all women of reproductive potential, before, during, and after pregnancy, as well as the urgent need to intensify measures to prevent unintended pregnancies.

According to data published in 2016, the cumulative vertical transmission rate by 18 months of age is 4.3%.^8^ The largest proportion (> 80%) of these transmissions occur during the first six months of the breastfeeding period,^8^ when women may experience pronounced challenges to adherence and retention in care, impacting negatively on viral suppression.^9,10,11,12^ At the same time, breastfeeding remains a key strategy to ensure that South African children survive and thrive. The evidence indicates that the benefits of breastfeeding outweigh the risks of not breastfeeding, regardless of the maternal HIV status.^13,14,15^ As the HIV epidemic matures, it is clear that the breastfeeding period must be one of the main priorities in the prevention of vertical transmission of HIV. New innovative strategies are required to achieve and maintain maternal viral suppression in the period after birth, whilst simultaneously promoting breastfeeding as a major child survival strategy. In addition, sustained maternal viral suppression will allow the realisation of the longer-term advantages of ‘Option B Plus,’ including improved maternal health, viral suppression in subsequent pregnancies, and reduced HIV transmission to sexual partners.

To this end, the South African Department of Health has revised the Guideline for the Prevention of Mother to Child Transmission of Communicable Infections (2019). This standalone guideline also forms part of the revised National Consolidated Guidelines for the Management of HIV in Adults, Adolescents, Children and Infants and for the Prevention of Mother-to-Child Transmission (2019). The guideline incorporates new evidence, both scientific and operational, to ensure that South Africa’s HIV PMTCT programme remains relevant, practical, and evidence-based. A concerted effort has been made to ensure alignment between these guidelines and other national guidelines, including the Standard Treatment Guidelines and Essential Medicines List for South Africa. It includes a strong focus on the prevention of HIV and unintended pregnancies in women of childbearing potential, maternal viral suppression, preventing MTCT during the breastfeeding period, and care integration for the mother-infant pair. A summary of the major changes in the guideline is illustrated in Table 1, together with the rationale for major changes for WLWH, being provided in the text.

**TABLE 1:** Summary of the main changes in the South African 2019 prevention of mother-to-child guideline.

Contents	2015 consolidated HIV guideline	2019 PMTCT guideline
Overall approach	-	A renewed focus on the practicality for the end user
		An operational focus on the integration of services, provision of a one-stop service, linkages to care, including care provided in the community by community health workers and other relevant stakeholders
Prevention	-	Guidance on universal infection precautions, and for preventing HIV acquisition in HIV-negative women and sero-discordant couples
Planned pregnancies and safe conception	-	Guidance for family planning and contraception in WLWH, as well as on safer conception
Maternal HIV testing	At first visit, at 32 weeks, and every three months during breastfeeding	At first visit and at every subsequent BANC visit, and three-monthly during breastfeeding
Maternal ART	-	Guidance on adherence messages
		Guidance for the use of DTG in women of childbearing potential
Maternal HIV VL monitoring	Guidelines for newly diagnosed mothers, and WLWH already on ART	Additional guidance for previously ART-exposed mothers
		VL done at delivery and at six months post-partum for all women, and six-monthly during breastfeeding
ART for the mother presenting in labour	Stat dose NVP and TDF/FTC, and AZT three-hourly during labour	Stat dose of NVP and a stat dose of TDF, 3TC, and DTG in a fixed-dose combination (TLD)
		Start ART next day (TLD preferred), after appropriate counselling
Infant HIV testing	HIV PCR test at birth and 10 weeks	HIV PCR test at birth and 10 weeks remain unchanged
		18-week HIV PCR test for high-risk infants removed
		Six-month HIV PCR test for all HIV-exposed infants introduced
	18-week PCR test for high-risk infants who received extended NVP for 12 weeks	
		At six months of age, establish the HIV status of all infants not already known to be HIV-exposed by offering an HIV test to the mother. If a maternal HIV test is not feasible, consent should be obtained to perform a rapid HIV test on the child.
	HIV test at six weeks post-cessation of breastfeeding	
		Age-appropriate HIV testing at six weeks post-cessation of breastfeeding is retained, with emphasis on testing even if breastfeeding continues for longer than 18 months
		18-month rapid test/ ELISA for all children regardless of HIV exposure (universal testing)
	18-month rapid test for HIV-exposed infants	
		HIV PCR test used as a confirmatory test for any HIV-positive result up to age two years
Definition of high-risk infant exposure	Maternal ART for < 4 weeks prior to delivery	High-risk infant at birth:
		Maternal VL ≥ 1000 copies/mL at delivery, or in the last 12 weeks of pregnancy
		No maternal VL result available in the last 12 weeks
		Unknown maternal HIV status because the infant is orphaned or abandoned
	Maternal VL ≥ 1000 copies/mL	
		High-risk infant during breastfeeding (> 72 h after delivery):
		New maternal HIV diagnosis during breastfeeding
		Maternal VL ≥ 1000 copies/mL after previous viral suppression on ART
Infant post-exposure prophylaxis	High-risk infants: AZT for six weeks and NVP prophylaxis for 12 weeks	AZT for six weeks and NVP prophylaxis for a minimum of 12 weeks. NVP is stopped after 12 weeks only if the maternal VL is proven to be < 1000 copies/mL. If the maternal VL is not suppressed by 12 weeks, continued NVP is given until maternal VL suppression is achieved, or until four weeks after breastfeeding cessation.
Breastfeeding	Breastfeeding recommended for 12 months (later changed to 24 months)	Breastfeeding recommended for 24 months or longer whilst ensuring maternal ART and viral suppression, in line with recommendations for the general population.

3TC, lamivudine; ART, antiretroviral therapy; AZT, zidovudine; BANC, basic antenatal care; DTG, dolutegravir; FTC, emtricitabine; HIV, human immunodeficiency virus; NVP, nevirapine; PCR, polymerase chain reaction; PMTCT, prevention of mother-to-child transmission of HIV; TDF, tenofovir; TEE, tenofovir, emtricitabine and efavirenz; VL, viral load; TLD, tenofovir, lamivudine and dolutegravir; WLWH, women living with HIV.

## Specific guidelines changes

### Antiretroviral therapy during pregnancy and the breastfeeding period

The risk of vertical HIV-transmission correlates strongly with maternal HIV VL levels.^16,17^ For every additional week on suppressive ART during antenatal care, the risk of MTCT is reduced by 10%.^2^ Therefore, the prescription of antiretroviral drugs that rapidly and safely achieve and sustain maternal viral suppression during pregnancy and the breastfeeding period is of greatest importance to the prevention of vertical transmission. In this regard, the newly introduced integrase inhibitor dolutegravir (DTG) offers improved tolerability, few drug interactions, and the reduced risk of viral drug resistance.^18^ The time to viral suppression is approximately halved by DTG when compared to the currently administered drug efavirenz (EFV).^18^

Recent data from Botswana indicates that DTG may increase the risk of neural tube defects (NTDs).^19^ The absolute risk is low, currently documented at 0.3% for mothers conceiving on a DTG-containing ART compared to a risk of 0.1% for mothers conceiving on alternative regimens.^20^ Whilst the World Health Organization (WHO) has recommended DTG as the preferred first-line option for all populations (weight ≥ 20 kg) without exceptions, South Africa has opted for a more conservative approach, and recommends that DTG should be used with caution in women wanting to conceive, and be avoided in the first six weeks of pregnancy, that is, following her last menstrual period and before closure of the foetal neural tube approximately four weeks after conception. However, recent evidence indicates that DTG is likely to have health and cost benefits over EFV even in women who intend pregnancy,^21^ providing further confirmation for the WHO’s more inclusive approach. Whilst South Africa’s position on DTG is likely to change as evidence evolves, confusion around the use of DTG in women of childbearing potential has resulted in the suboptimal uptake of effective ART in the women who need it most.

As a positive consequence, the current DTG recommendations will require that family planning services be better integrated into ART care. Health care workers should regularly discuss issues of childbearing and contraception with their clients in order to understand current fertility intentions and contraceptive needs. All women require appropriate counselling on the risks and benefits of DTG and should make an informed choice (Box 1). Women may choose to use DTG; for those women who choose not to use DTG, EFV remains a safe, efficacious and cost-effective option. Concurrent use of effective contraception is recommended for all non-pregnant women not currently desiring a pregnancy.

BOX 1 Use of dolutegravir in women of childbearing potential.

Care should be provided in ways that respect women’s autonomy in decision-making about their health, and services must provide information and options to enable women to make *informed choices*.^22^ To this end, all women considering the use of dolutegravir (DTG) should be informed of the following:

- Dolutegravir is an effective and well tolerated antiretroviral drug that is safe to use during pregnancy after closure of the foetal neural tube (from the seventh week of pregnancy onwards). Current evidence indicates that DTG is safe to use during lactation.^23,24^
- Using DTG at the time of conception carries an approximately 0.3% risk of a neural tube defect (NTD) in the infant, according to currently available evidence. This compares to a 0.1% background risk of having a child with a NTD if an alternative regimen is used.^20^
- It is advised that any non-pregnant woman using DTG should use reliable and effective contraception (hormonal methods or an intra-uterine contraceptive device). On subsequent changes in a woman’s fertility intentions, coupled with concerns about the risk of NTDs, a switch from TLD to TEE may be offered, provided a suppressed HIV viral load in the last six months has been documented.
- Women already on antiretroviral therapy wanting to switch to a DTG-containing regimen should be aware of the following in addition to the points listed above:
  ■ New side effects may be experienced when switching from an EFV-containing regimen to a DTG-containing regimen (insomnia, headache or gastro-intestinal disturbances). These are uncommon, usually mild and self-limiting, and should be reported to the health care provider immediately without stopping treatment.
  ■ Efavirenz-based regimens should be taken at night, whilst the DTG-based regimens can be taken either in the morning or at night. If insomnia is experienced, morning dosing might be preferable.

DTG, dolutegravir; NTD, neural tube defects; EFV, efavirenz; TLD, tenofovir, lamivudine and dolutegravir; TEE, tenofovir, emtricitabine and efavirenz.

### Regimen switches during pregnancy

A single drug switch from an EFV-containing regimen to a DTG-containing regimen should only be considered if the client has a suppressed VL (in the last six months), irrespective of pregnancy status. Therefore, pregnant women already on ART should continue their current ART regimen, pending the result of their HIV VL at entry into antenatal care. If the VL is below 50 copies/mL, and the woman has progressed past the initial six weeks of pregnancy, that is, six weeks since her last normal menstruation, switching to a DTG-containing regimen may be offered. Appropriate counselling as outlined in Box 1 should precede the use of DTG in any women of childbearing potential.

### Antiretroviral therapy for women with previous antiretroviral exposure

Women presenting during pregnancy and breastfeeding who are not on ART but with previous exposure to ART present a unique dilemma, given the urgency for maternal VL suppression. They may already have developed viral resistance, with the potential of delayed viral suppression on the re-starting of EFV-containing regimens. For this reason, women known to be living with HIV who are currently not on ART, but who are ART-exposed, for example previous PMTCT, or previous loss-to-follow-up on ART, should initiate a DTG-containing regimen. The fixed-dose combination of tenofovir (TDF), lamivudine (3TC), and DTG, known as TLD, is recommended with previously documented VL suppression on ART. Without proof of prior viral suppression (with previous VL results either not suppressed or not available), zidovudine (AZT), 3TC and DTG should be initiated, due to the higher likelihood of concomitant resistance to both the TDF and EFV components of their first-line fixed-dose combination regimen.

### Antiretroviral therapy for women presenting in labour

For women initiating ART around the time of labour and delivery, earlier guidelines recommended one dose of nevirapine (NVP) and TDF/emtricitabine (FTC), together with three-hourly AZT during labour.^25^ This has been replaced with a single dose of NVP, together with a dose of TLD (See Table 1, row 7: ART for the mother presenting in labour). The dose of NVP is retained due to existing evidence.^26^ Previously, in the era where not all women were eligible for ART post-delivery, the TDF/FTC combination was used to ‘cover the tail’ and reduce the risk of viral resistance. However, all women now qualify for ART. The TLD given in labour provides the first dose of lifelong ART and removes the need for both TDF/FTC and three-hourly AZT during labour. Antiretroviral therapy is to be continued the following day after understanding the woman’s fertility intentions and appropriate counselling on the risk of DTG-associated NTDs for her subsequent pregnancies. The recommended regimen in the period after birth is TLD. Concurrent use of effective contraception is recommended for all women at discharge from labour wards.

**TABLE 2:** Infant HIV post-exposure prophylaxis given at birth.

Risk category	Indications	Infant prophylaxis
Low-risk infant at birth, whether breastfed or exclusively formula fed	Maternal VL at delivery < 1000 copies/mL	Infant NVP at birth and then daily for six weeks
High-risk infant at birth in the breastfed infant	Mother not on ART at delivery, or	Infant NVP for a minimum of 12 weeks. Infant NVP only discontinued after confirmation of maternal VL being < 1000 copies/mL, and or until four weeks after cessation of all breastfeeding.
	Mother on ART with HIV VL ≥ 1000 copies/mL at delivery, or prior to 12 weeks	
	No HIV VL result available at delivery or prior to 12 weeks	Infant AZT twice daily for six weeks
High-risk infant at birth in the infant exclusively formula fed from birth	Mother not on ART at delivery, or	Infant NVP at birth and then daily for six weeks (provided that avoiding breastfeeding is documented and sustained), and
	Mother on ART with HIV VL ≥ 1000 copies/mL at delivery, or prior to 12 weeks	
		Infant AZT twice daily for six weeks
	No HIV VL result available at delivery or prior to 12 weeks	

ART, antiretroviral therapy; AZT, zidovudine; NVP, nevirapine; VL, viral load; HIV, human immunodeficiency virus.

### Maternal human immunodeficiency virus viral load monitoring

Potent ART regimens are most effective when coupled with HIV VL monitoring to confirm good adherence and viral suppression or to allow the timely detection of factors that may negatively affect viral suppression (e.g. suboptimal treatment adherence, drug interactions, intercurrent infections, etc.). Importantly, VL monitoring can only be effective if coupled with a *response* to the test results, and women with detectable VL results (VL > 50 c/mL) must be followed up for urgent intervention, directed to ensure immediate viral suppression. Accurate national surveillance of maternal VL suppression will require a means to distinguish between VLs done during antenatal care, at delivery, and after birth. Within the public sector this will be achieved through the use of electronic gatekeeping codes submitted on National Health Laboratory Service (NHLS) requisition forms (code *C#DELIVERY* applied to VLs at delivery, and *C#PMTCT* applied to VLs during the antenatal and postpartum periods). Of great importance, colleagues in the private sector may wish to discuss with their laboratory service the value or feasibility of implementing such a coding system in their practice.

### Viral load monitoring during the antenatal period

Women initiating or re-initiating ART in pregnancy should have their first VL measured three months after starting ART. If suppressed, the VL should be repeated at delivery. For WLWH and who are already on ART, the VL should be measured at the first antenatal care visit, as per previous guidelines. If suppressed, the VL is repeated at delivery, unless subsequent VL non-suppression is suspected. Accurate recording, of antenatal maternal VL testing data in the maternity case record, is crucial to ensure communication and continuity of care between antenatal and delivery services.

Women initiating ART at any time after 28 weeks gestation will have a VL measurement done at delivery and repeated three months after delivery. If the mother-infant pair is receiving integrated care, this VL should be aligned to the 10-week well-child immunisation visit.

### Viral load monitoring at delivery and six months after delivery

Previous implementation of VL monitoring in the PMTCT programme has been suboptimal. Thus, the VL monitoring schedule, that had been linked to the ART history and timing of ANC booking, has been changed to fixed time points linked to the pregnancy itself. A VL at delivery has, therefore, been introduced for all WLWH. Much debate has ensued with regard to the best timing of a VL around the time of delivery. Whilst results of a VL done at 36 weeks’ gestation would be available by the time of delivery, a significant proportion of women, including those with premature labour, those with uncertain gestational age, and un-booked deliveries, would not receive a VL at 36 weeks’ gestation. Additionally, antenatal care usually occurs at a different facility from delivery, and antenatal VL results may not be available at the delivery site.

A VL done at the actual time of delivery has the following advantages:

- High in-facility birth rates, and previous rapid uptake and high coverage of infant birth PCR testing within labour wards, infer the possibility of near-universal coverage of the delivery VL.
- Uniformly performed VLs at time-point of delivery will enable the health system to monitor and better enforce the coverage of maternal VL testing.
- Viral load testing at delivery allows for placing of the reference to the maternal laboratory testing into the infant’s Road-to-Health Booklet to provide a potential link for PMTCT interventions, promoting integrated care for the mother-infant pair and ensuring consideration of maternal laboratory results during infant health care provision.
- It provides a means of epidemiological surveillance of vertical transmission risk at delivery.

Given that more than half of vertical transmissions now occur after delivery,^8,27^ linked to postpartum challenges to adherence and retention in care,^9,10,11,12^ a maternal VL should be done at six months after delivery for all WLWH, aligned to the six-month well-child immunisation visit, regardless of breastfeeding status. As the maternal VL impacts directly on the care required for the infant, breastfeeding mothers who are not receiving integrated care as a mother-infant pair and who are receiving care at a separate ART clinic face a significant challenge. General ART services have, to date, not effectively identified breastfeeding mothers, nor monitored their VLs at six-monthly intervals. If breastfeeding is to be protected by providing enhanced infant antiretroviral prophylaxis in mothers who have detectable VLs, much will need to be done to improve the identification of breastfeeding mothers and their infants, VL monitoring, and communication with providers caring for their infants.

Clinical interventions alone will not improve postpartum viral suppression. Health workers, supported by community engagement and media campaigns, should empower mothers with knowledge regarding the importance of continued viral suppression for both maternal and child health benefits. Women need to be equipped to anticipate adherence challenges in the postpartum period, including disrupted routines, sleep deprivation, and postnatal depression. Linkages to available resources should occur, for example community health workers, mentor mothers, MomConnect, or other support groups and clubs. A concerted effort should be made by all in the care team to ensure that the mother is retained in care, adherent to ART, maintains VL suppression and has access to effective contraceptive services.

### Management of an elevated maternal human immunodeficiency virus viral load

Viral load suppression across the South African ART programme is now defined as a VL of < 50 copies/mL. Any VL ≥ 50 copies/mL implies viral replication and risk to the mother and infant. If the mother is on ART and her VL is detectable, drug resistance must be considered. But such a situation needs to trigger a thorough assessment, for example evaluation of adherence, a check of medication dosages, a review of potential drug-drug interactions and the exclusion of intercurrent infection. A cause needs to be identified and corrected. An elevated maternal VL of ≥ 1000 copies/mL at delivery or during the breastfeeding period warrants initiating, extending, or re-starting high-risk infant ART prophylaxis (see below).

The threshold for virological failure and switch to second-line ART is influenced by the treatment regimen, namely an EFV or a DTG-containing regimen, and the prior duration of ART. *The definition of virological failure whilst on an EFV-based regimen remains a VL of* ≥ *1000 c/mL on two consecutive occasions* ideally not longer than three months apart, despite attention to adherence issues. The definition of failure on a DTG-based regimen requires at least three VLs ≥ 1000 copies/mL over a two-year period together with a focused effort to improve the patient’s adherence. Women failing to suppress on second- or third-line ART warrant expert discussion or referral. These clients are likely to be experiencing complex clinical and psychosocial challenges and will likely benefit from an individualised approach to maternal management, infant prophylaxis, and recommendations for possible breastfeeding cessation and the prescription of infant formula.

### Infant post-exposure prophylaxis to be provided at birth

The delivery VL will determine the infant’s HIV transmission risk category. However, many mother-infant pairs will be discharged after birth but before the delivery VL results are available. Then the most recently available VL result, that is, within the last 12 weeks, will determine the infant’s risk category prior to discharge at birth. The laboratory barcode sticker or similar indicator of the delivery VL, clearly labelled ‘maternal’ to distinguish it from the infant’s HIV PCR barcode sticker, should be placed in the infant’s patient records, together with the Road-to-Health Booklet, to ensure access to both maternal and infant birth-PCR results at the 3–6-day postnatal visit. Adjustments can then also be made to the infant prophylaxis regimen, as appropriate.

- *Low-risk infants*, namely infants born to mothers with a suppressed VL within the last 12 weeks of delivery, or at delivery, should receive NVP daily for six weeks.
- *High-risk infants* identified at birth, namely no recent VL available in the last 12 weeks of pregnancy or maternal VL ≥ 1000 copies/mL, should receive high-risk prophylaxis: NVP daily for a minimum of 12 weeks and AZT twice daily for six weeks.
- *A persistently elevated maternal VL in the breastfeeding period* is a further risk for HIV acquisition in the infant.^16,17^ For this reason, NVP should only be stopped after 12 weeks post-delivery if the maternal VL is < 1000 copies/mL. Otherwise NVP should be continued until a maternal VL of < 1000 copies/mL is achieved or until four weeks after breastfeeding cessation.

Table 2 provides a summary of infant post-exposure prophylaxis to be given at birth, based on the infant’s risk profile for HIV transmission and chosen feeding method.

### Infant human immunodeficiency virus testing

Infant HIV PCR testing at birth is recommended in all HIV-exposed infants, to identify intra-uterine transmission. Wherever possible, birth HIV PCR tests should not be submitted with the mother’s name and details such as ‘Baby of’. Mothers should be counselled during antenatal care to provide the permanent name and surname of the child at delivery. Correct identification information for the child will allow the birth PCR to be linked to later test records, and will facilitate registration of the birth with home affairs before discharge.

HIV PCR testing at age 10 weeks is recommended to identify perinatal HIV transmission. This remains part of the standard schedule of testing for HIV-exposed infants (Table 3). However, the HIV PCR test at 18 weeks, previously performed in infants who received high-risk prophylaxis, has been discontinued due to inadequate uptake and non-alignment with any child immunisation visit. Instead, all HIV-exposed infants, both low and high risk, are to receive an HIV PCR test at the six-month integrated well-child visit. Over 80% of infants who acquire HIV do so by six months of age, highlighting the importance of this six-month HIV PCR test.^8^ However, the risk of infant HIV acquisition may shift to more than 20% after six months of age if longer periods of breastfeeding are achieved. Breastfed infants who test HIV negative, particularly those on antiretroviral prophylaxis, should not be assumed to be uninfected until after the age-appropriate post-weaning HIV test has been performed.

**TABLE 3:** Time points for routine HIV testing in HIV-exposed and HIV-unexposed children.

Timing and type of HIV test	HIV-exposed infants	HIV-unexposed infants
HIV PCR test at birth	x	-
HIV PCR test at age 10 weeks	x	-
HIV PCR test at age six months (offer HIV testing for documented HIV negative mothers or mothers with unknown HIV status)	x	-
Age-appropriate HIV test at six weeks post-cessation of breastfeeding	x	-
HIV antibody test at age 18 months (rapid test or HIV ELISA) (universal testing)	x	x
Age-appropriate HIV test at anytime the child is unwell	x	x
If any HIV test is positive, the diagnosis is confirmed with a repeat HIV test (see guidelines below in Table 4) , and ART is initiated immediately.	x	x

ART, antiretroviral therapy; ELISA, enzyme-linked immunosorbent assay; HIV, human immunodeficiency virus; PCR, polymerase chain reaction; VL, viral load.

At six months of age, the HIV status of all infants not already known to be HIV-exposed should be established by offering an HIV test to the mother. This maternal HIV test should fall into the routine three-monthly HIV testing schedule for all breastfeeding mothers who are not yet known to be living with HIV. If a maternal HIV test is not feasible, consent should be obtained to perform a rapid test on the child. Care that is provided in an integrated manner to the mother-infant pair greatly facilitates this process of (1) identifying a women as breastfeeding, (2) identifying those women who require HIV testing, and (3) determining the care required by the infant as informed by the mother’s results. Breastfeeding mothers who are not receiving integrated care face a significant challenge with regard to repeat HIV testing. Much will need to be done to ensure that breastfeeding mothers are able to access HIV testing services within family planning clinics and other general health services, and that her infant receives the appropriate care according to her test results.

An HIV antibody test (HIV rapid or ELISA [enzyme-linked immunosorbent assay] test) is used as a screening test above 18 months of age. However, in a small percentage of children the maternal antibodies persist beyond 18 months of age, potentially resulting in false-positive HIV diagnoses and inappropriate initiation of lifelong ART.^22^ Therefore, HIV PCR testing is now recommended as confirmatory testing in all HIV-positive HIV rapid or ELISA tests in children under two years of age. A summary of initial and confirmatory HIV testing is outlined in Table 4.

**TABLE 4:** HIV tests for initial and confirmatory testing in children according to age: 2019 prevention of mother-to-child transmission guidelines.

Age of child	Initial HIV test	Confirmatory HIV test
Below 18 months of age	HIV PCR test	HIV PCR test (or VL test)
18–24 months	HIV Rapid/ELISA test	HIV PCR test
Two years and older	HIV Rapid/ELISA test	HIV Rapid/ELISA test

ELISA, enzyme-linked immunosorbent assay; HIV, human immunodeficiency virus; PCR, polymerase chain reaction; VL, viral load.

At the clinician’s discretion, the HIV PCR may be replaced by a VL test, which has the advantage of both confirming the HIV diagnosis and providing a baseline VL for monitoring the child’s response to ART. Diagnostic difficulties, for example indeterminate HIV PCR test results, require urgent expert advice.

## Breastfeeding

Due to the expanded access to ART in South Africa, the country-level recommendations for breastfeeding are now the same whether or not the mother is living with HIV.^15,28^ These recommendations include exclusive breastfeeding for the first six months, the introduction of appropriate complementary foods thereafter, and continued breastfeeding for two years or longer. Given the numerous benefits of breastfeeding for the health and well-being of all children, it is imperative that mothers are supported to breastfeed their infants for the longest possible duration whilst maintaining viral suppression and reducing the risk of HIV-transmission through breastmilk exposure.

*To reduce HIV transmission during breastfeeding*, the guideline outlines two major strategies. The first is to improve viral suppression rates in the period after birth by (1) providing potent and well-tolerated ART regimens – including DTG, (2) outlining mechanisms for linking mothers back into appropriate HIV care post-delivery, (3) integrating services for mother-infant pairs to promote adherence and retention in care, and (4) using VL monitoring strategically for the timely detection of, and response to, elevated HIV VLs. Whilst maternal viral suppression remains the gold standard, elevated HIV VLs will occur in selected women in the breastfeeding period. The second strategy is to provide enhanced infant prophylaxis whilst every effort is made to regain maternal viral suppression.

### Prophylaxis for infants of breastfeeding mothers with an elevated human immunodeficiency virus viral load

An elevated maternal HIV VL during breastfeeding can occur either at the time of a new HIV diagnosis or because of an unsuppressed VL on ART. Regardless of the cause, an elevated HIV VL in a breastfeeding mother requires urgent clinical intervention and more frequent VL monitoring. With pre-treatment resistance to non-nucleoside reverse transcriptase inhibitors documented to be on the increase, and the high likelihood (up to 40%) of viral resistance in the mother with an elevated VL on ART,^29,30^ these infants should receive high-risk prophylaxis consisting of AZT twice daily for six weeks, and NVP daily for a minimum of 12 weeks, with infant NVP only stopped after confirmed maternal HIV VL < 1000 c/mL, or breastfeeding cessation.

Consolidated HIV PCR and VL Results for Action reports, generated daily or weekly from the NHLS data warehouse, can be used to fast track high-risk clients, including pregnant women with high VLs and HIV-infected infants. Reports for the public sector can be accessed by registering at http://www.nicd.ac.za.

## Conclusions

Whilst much progress has been made in preventing vertical transmission of HIV, much remains to be done. Overcoming the ‘next frontier’ and preventing vertical HIV transmission during the breastfeeding period may well be the most challenging phase yet. In response, the new 2019 PMTCT guideline outlines strategies that focus strongly on maintaining maternal HIV viral suppression, strengthening and integrating care for mother-infant pairs, and supporting and protecting breastfeeding as a major child survival strategy. Creating an environment that enables the rigorous implementation of this guideline will move South Africa closer to the goal of eliminating vertical HIV transmission and making an HIV-free generation a reality.

The Guideline for the Prevention of Mother to Child Transmission of Communicable Infections is available at http://bit/ly/2019-PMTCT-Guidelines.
